# Laparoscopic liver resection for hepatocellular carcinoma in Fontan-associated chronic liver disease. The first case report

**DOI:** 10.1016/j.ijscr.2019.05.029

**Published:** 2019-05-23

**Authors:** Roberta Angelico, Veronica Lisignoli, Lidia Monti, Rosanna Pariante, Chiara Grimaldi, Maria Cristina Saffioti, Maria Giulia Gagliardi, Marco Spada

**Affiliations:** a Division of Abdominal Transplantation and Hepatobiliopancreatic Surgery, Bambino Gesù Children’s Hospital IRCCS, Rome, Italy; b Department of Cardiology, Division of Grow Up Congenital Heart, Bambino Gesù Children’s Hospital IRCCS, Rome, Italy; c Department of Radiology, Bambino Gesù Children’s Hospital IRCCS, Rome, Italy; d Department of Anesthesiology, Bambino Gesù Children’s Hospital IRCCS, Rome, Italy

**Keywords:** AFP, alphafetoprotein, CLD, chronic liver disease, CT, computer tomography, FP, Fontan procedure, HCC, hepatocellular carcinoma, LLR, laparoscopic liver resection, RFA, radiofrequency ablation, Hepatocellular carcinoma, Fontane procedure, Laparoscopic surgery, Liver resection, Chronic liver disease, New technology

## Abstract

**Introduction:**

A well-recognized long-term complication after Fontan procedure (FP), a complex cardiac surgery performed in patients with univentricular hearts, is the development of chronic liver disease and hepatocellular carcinoma (HCC). Due to the risk of cardiac and liver decompensation, liver resection of HCC is challenging and the laparoscopic approach has never been reported.

**Presentation of the case:**

We present the first case of laparoscopic liver resection (LLR) of HCC in a 33-years-old girl with cardiac-related cirrhosis after FP. Intraoperatively, the pneumoperitoneum was established at 8–10 mmHg and adequate fluid infusion was given to maintain the cardiac preload. After an ultrasound-guided thermoablation along the free-tumor margin of the hepatic lesion, a full laparoscopic non-anatomical resection of the tumor in segment V was performed, without Pringle manouver and blood transfusion requirement. The cardiac function remained stable during the surgery and thereafter, and the post-operative course was uneventful.

**Discussion:**

HCC in chronic liver disease after FP is associated with high-risk mortality. Due to the complex hemodynamic changes after FP, open surgical resections often aren’t feasible and loco-regional percutaneous treatment or combined liver-heart transplantation are the only therapeutic options. This case suggests that LLR in FP patients has low-risk of liver and cardiac decompensation, minimizing the pneumoperitoneum insufflation to ensure low intra-abdominal/intra-thoracic pressures and providing accurate anaesthetic management to maintain proper cardiac preload and output.

**Conclusion:**

LLR for HCC after FP is safe and feasible, and might be considered an alternative treatment of HCC for which the best treatment has not been defined yet.

## Highlights

• Hepatocellular carcinoma after Fontan procedure is associated with high mortality.
• Liver resection after Fontan procedure has high-risk liver/cardiac decompensation.
• Laparoscopic liver resection is feasible with low intra-abdominal pressures.
• Adequate anaesthetic management is essential in Fontan procedure patients.
• Laparoscopic liver resection is a new therapeutic option after Fontan procedure.

## 1 Introduction

Hepatocellular carcinoma (HCC) associated with chronic liver disease (CLD) is an increasing recognized complication after Fontan procedure (FP) due to long-term complex hemodynamic changes [1]. The FP is a palliative cardiac surgery used to divert the systemic and pulmonary vascular circulation in children with single ventricle physiology, whose survival dramatically increased over 80% after 20 years [1]. In the Fontan circulation, the systemic venous return is sent to the pulmonary arteries bypassing the right ventricle, with consequent chronic venous congestion causing CLD [1]. HCC might occur late after FP (>10 years) and is associated with high mortality [2]. Surgery represents the only curative treatment for HCC > 3 cm, however hepatic resections are challenging after FP for complex cardiac circulation and the CLD background, thus combined liver-heart transplantation often remains the only therapeutic option [3].

Despite the known benefits of laparoscopic liver resection (LLR) in CLD [4], the laparoscopic approach has never been reported after FP. The limitations of LLR in Fontan-associated liver disease are related firstly to the adverse effects of pneumoperitoneum on the Fontan circulation due to increasing intra-abdominal and intra-thoracic pressure, rising of pulmonary and systemic resistance, and reduction of the cardiac preload and output, which could be fatal [5]; secondly, a severe portal hypertension may cause high-risk of bleeding during liver resection [3]. To the best of our knowledge, we report the first case of LLR for HCC after FP. The work has been reported in line with the SCARE criteria [6].

## 2 Case presentation

**Fig. 1:**
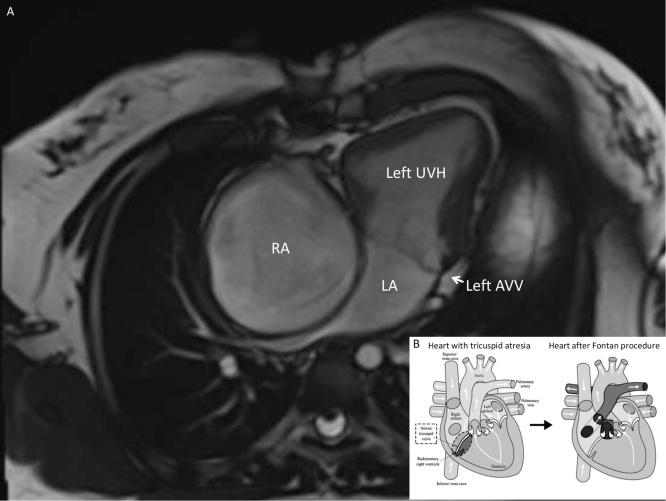
**Cardiac imaging and physiopathology after Fontan procedure**. Cardiac magnetic resonance (CRM) and schematic diagram of heart physiopathology in patient with tricuspid atresia after Fontan procedure. A) CRM cine_4 chamber view in a 33-years old girl born with tricuspid atresia who underwent palliative direct atrio-pulmonary connection (Fontan procedure) at 6 years of age; B) Schematic diagram of the heart with tricuspid atresia before and after Fontan procedure (copyright license defined by the GNU Free Documentation License, source: https://commons.wikimedia.org/wiki/File:Fontan_procedure.sv). ***Abbreviations:****AVV: atrio-ventricular valve; CRM,* Cardiac magnetic resonance; *LA: left atrium; RA: right atrium; UVH: univentricular heart*.

**Fig. 2:**
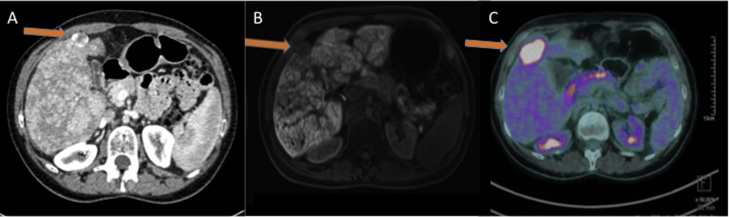
**Imaging of hepatocellular carcinoma after Fontan procedure**. Pre-operative images in a 33-years old girl with hepatocellular carcinoma and chronic liver disease after Fontan procedure. A) Computer tomography scan showing hypervascular lesion in segment V on arterial phase; B) Axial fat-suppressed T_1_-weighted Magnetic resonance imaging with gadoxetic acid administration obtained at 20 min, hepatocellular phase, detecting strong enhancement of the background liver parenchyma, but no uptake in the hepatocellular carcinoma; C) Fluorine-18 fluorodeoxyglucose (FDG) positron emission tomography showing increased FDG uptake in the hepatic lesion.

A 33-year-old female, who had undergone FP as palliation for a single ventricle anomaly at 6 years of age (Fig. 1), presented alphafetoprotein (AFP) of 3005 ng/mL. Computer Tomography (CT) showed features of CLD associated with a 3.4 x 4.5 cm solid hepatic lesion in segment V (Fig. 2). Considering the compensated cardiocirculatory condition (normal systolic heart function, no arrhythmias, good functional capacity) and the CLD grading (Model for End-Stage Liver Disease score: 9, Child-Turcotte-Pugh stage: A) a LLR was planned.

### 2.1 Surgical procedure

**Fig. 3:**
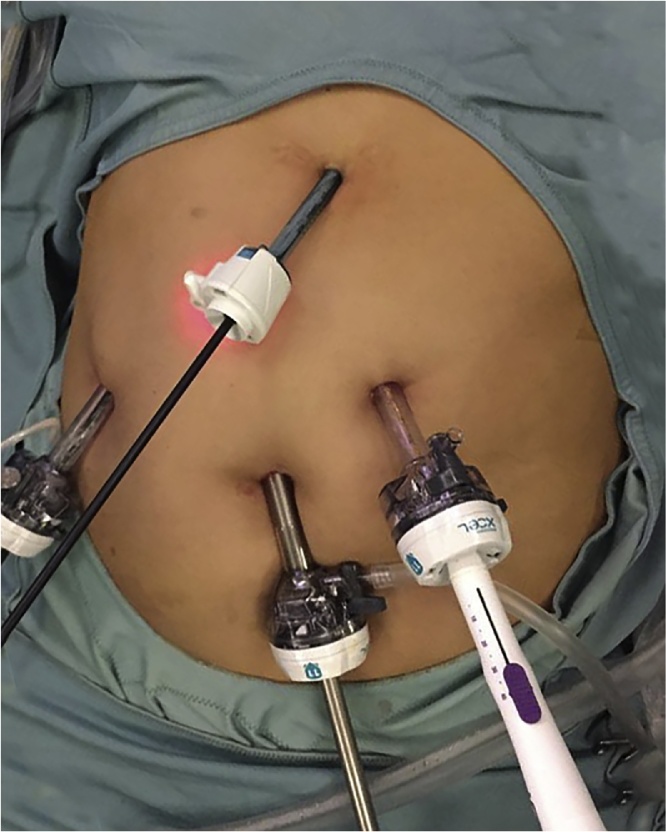
**Trocar position for laparoscopic liver resection**. Surgery was performed by the insertion of four trocars: one sovraumbelical for the camera (11 mm), one working trocar in right lateral flank (11 mm), one working trocar in left pararectal position (12 mm) (from which the laparoscopic ultrasound probe was inserted) and one auxiliary trocar in the epigastric region (5 mm). The auxiliary epigastric trocar was inserted to use the aspirator during the liver resection, due to the high risk of bleeding from the cirrhotic liver, and to facilitate the laparoscopic cholecystectomy by holding the fundus of the gallbladder as for standard manner (the cholecystectomy was required due to its close proximity to the hepatic tumor).

The patient was placed in supine position, with her legs apart to apply the French position and the surgeon stood between the patient’s legs. Four trocars (two 11 mm, one 12 mm, one 5 mm) were placed into the abdomen as shown in Fig. 3. After achieving a predetermined pressure of 10 mmHg, a 30-degree endoscope was inserted and a cirrhotic liver with small amount of ascites was visualized. The intraoperative ultrasound of the liver confirmed a 4 x 4.5 cm lesion of segment V near by the gallbladder and multiple regenerative nodules not suspicious of malignancy. Due to the close proximity of the lesion to the gallbladder, a conventional laparoscopic cholecystectomy was carried out.

With the assist of intraoperative ultrasound, an inner line was made on the liver surface with diathermy to mark the periphery of the tumor. Then, a radiofrequency ablation (RFA) with single needle probe was performed along the free-tumor margin of the hepatic lesion (2 cm outside -away from- the inner line of the tumor) in order to reduce the cut surface bleeding. The next step was an non-anatomical wedge resection of segment V. The parenchymal transection was performed without Pringle manoeuvre, by applying the harmonic scalper and the Cavitron Ultrasonic Surgical Aspirator (CUSA). Vessel structures were clipped by locked clips and then cut. The resected lesion and the gallbladder were extracted by endobag trough the supraomeblical trocar incision. The pneumoperitoneum was maintained at 8–10 mmHg through out the entire procedure and intraoperative blood losses were 100 mL.

During surgery, the anaesthetic management consisted of sevuforane/fentanyl and invasive central venous/arterial pressures monitoring to maintain a stable cardiac function and oxygenation, which were achieved by infusion of intravenous fluids (11 ml/Kg/h) and dopamine (3 mcg/Kg/min). A stable cardiac function was maintained during all intra- and peri-operative phases.

### 2.2 Histological findings

A well-differentiated HCC with trabecular growth pattern (Edmondson grade I; absence of vascular infiltration) of 4.5 × 4 x 4 cm (volume: 72 cm^3^). No malignant cells were found in surgical margins of 1 cm (R0 resection). For immunohistochemistry, the HCC showed positive glypican, while negative pattern for beta-catenine, glutamine synthetase and loss of expression of liver fatty acid binding protein.

### 2.3 Post-operative course

Post-operative course was uneventful, characterized by stable liver and cardiac function; after 7 days the patient was discharged. After 3 months, AFP level was 30.14 ng/mL with negative CT. At 7 months from surgery HCC recurrence was detected and Sorafenib treatment combined with transarterial radioembolization was performed as downstage for combined heart-liver transplantation.

## 3 Discussion

In Fontan-associated CLD the management of HCC isn’t defined yet due to its complexity and rarity [3]. The few cases reported in literature (n = 18) don’t allow drawing guidelines yet. The current treatment options for HCC in Fontan patients with CLD include: open liver resection, associated with high risks of bleeding and post-operative liver-decompensation (including coagulopathy and ascites) causing severe cardiac dysfunction; chemoembolization or radiofrequency ablation, which frequently aren’t feasible due to the presence of cardiac pacemakers, extrahepatic shunts and risk of thromboembolism; therefore combined liver-heart transplantation is often the only curative alternative [3].

This case suggests that LLR in FP patients has lower risk of blood loss and liver decompensation, as demonstrated in patients with cirrhotic liver from other diseases [4]. The laparoscopic approach might represent a feasible treatment in patients with Fontan physiology by 1) minimizing the pneumoperitoneum insufflation to ensure low intra-abdominal/intra-thoracic pressures and satisfactory ventilation, with low pulmonary/systemic resistance; 2) adequate intravascular volume administration to maintain good cardiac output; 3) avoiding surgical techniques that reduce the cardiac preload (Pringle manoeuvre and blood losses during parenchymal transection).

The liver resection assisted by RFA is a useful tool in Fontan patients with cirrhotic liver since RFA provides an avascular liver resection plan, minimizing blood loss and the need of hepatic inflow obstruction (Pringle manoeuvre). Moreover, the RFA along the cut surface is effective in parenchymal sparing and in achieving complete tumor resection (R0) as demonstrated in other series [7].

## 4 Conclusion

LLR for HCC in FP patients is safe and feasible thus it needs an adequate pre-operative assessment of the cardiac function and the CLD severity. In FP patients, the LLR might be considered as an alternative approach offering potential short- (decreased cardiac-liver decompensation, early recovery) and long-term (reduced visceral adhesions) benefits, either as definitive or bridge treatment before transplantation.

## Conflicts of interest

All authors have no conflict of interest.

## Sources of funding

All authors didn’t receive any financial support for the manuscript.

## Ethical approval

The study has been exempt from ethical approval by out institution.

## Consent

Written informed consent was obtained from the patient for publication of this case report and accompanying images. A copy of the written consent is available for review by the Editor-in-Chief of this journal on request

## Author contribution

Roberta Angelico: wrote and desiged the paper, analyzed and interpreted data, approved the final version, reviewed the accuracy and integrity of the work.

Veronica Lisignoli: collected data, analyzed and interpreted data, approved the final version, reviewed the accuracy and integrity of the work.

Lidia Monti: analyzed and interpreted data, approved the final version, reviewed the accuracy and integrity of the work.

Rosanna Pariante: analyzed and interpreted data, approved the final version, reviewed the accuracy and integrity of the work.

Chiara Grimaldi: analyzed and interpreted data, approved the final version, reviewed the accuracy and integrity of the work.

Maria Cristina Saffioti: analyzed and interpreted data, approved the final version, reviewed the accuracy and integrity of the work.

Maria Giulia Gagliardi: designed the work, analyzed and interpreted data, approved the final version, reviewed the accuracy and integrity of the work.

Marco Spada: performed the surgery, designed the work, analysed and interpreted data, revisited critically the manuscript for intellectual content, approved the final version, reviewed the accuracy and integrity of the work.

## Registration of research studies

Not required.

## Guarantor

Marco Spada, MD, PhD, FEBS.

## Provenance and peer review

Not commissioned, externally peer-reviewed.
